# Clinical features and chest CT findings of *Chlamydia pneumoniae* pneumonia

**DOI:** 10.3389/fmed.2026.1717744

**Published:** 2026-01-27

**Authors:** Haoyu Zheng, Xuelei QuBie, Jin Wang, Pingping Liu, Wei Zhang

**Affiliations:** Department of Radiology, Sichuan Provincial Corps Hospital, Chinese People’s Armed Police Forces, Leshan, China

**Keywords:** *Chlamydia pneumoniae*, computed tomography, imaging pattern, metagenomic next-generation sequencing, pneumonia

## Abstract

**Objective:**

This study aimed to investigate the clinical features and chest computed tomography (CT) findings in 42 patients with *Chlamydia pneumoniae* pneumonia, as confirmed by metagenomic next-generation sequencing (mNGS).

**Methods:**

We conducted a retrospective analysis of clinical data and chest CT findings (both at disease onset and within 1 month thereafter) in 42 patients diagnosed with *Chlamydia pneumoniae* pneumonia by mNGS at our hospital between August 2022 and August 2025.

**Results:**

Of the 42 patients, 25 (59.5%) presented with fever, 26 (61.9%) with sore throat, 30 (71.4%) with cough, 27 (64.3%) with expectoration, 11 (26.2%) with myalgia, 10 (23.8%) with general fatigue, and 10 (23.8%) with neurological symptoms such as headache and dizziness. Laboratory tests revealed that 12 patients (28.6%) showed a mild increase in white blood cell count, 10 (23.8%) had elevated neutrophil counts, 21 (50.0%) exhibited elevated C-reactive protein (CRP) levels, and 6 (14.3%) had CRP levels exceeding 100 mg/L. In the early stage, chest CT demonstrated a lobular pneumonia pattern in 16 patients (55.2%), involvement of a single lung lobe in 20 (69.0%), predominant lower-lung distribution in 19 (65.5%), and a nodular-patchy pattern in 8 patients (27.6%) with a nodular–patchy pattern. The main accompanying features included a halo sign in 25 patients (86.2%), centrilobular nodules in 23 (79.3%), and bronchial wall thickening in 20 (69.0%). In the mid-to-late stage, chest CT revealed a lobular pneumonia pattern in 23 patients (76.7%), single-lobe involvement in 23 (76.7%), and predominant lower-lung distribution in 20 (66.7%). The major concomitant features were a halo sign in 21 patients (70.0%), centrilobular nodules in 20 (66.7%), and bronchial wall thickening in 24 (80.0%).

**Conclusion:**

Chest CT findings of *Chlamydia pneumoniae* pneumonia are predominantly characterized by a lobular pneumonia pattern, lower-lobe distribution, and associated features such as bronchial wall thickening, centrilobular nodules, and a peripheral halo sign. Certain imaging differences exist between early and middle-to-late stages, with the nodular-patchy pattern potentially representing an ultra-early imaging marker, which may provide clues for early clinical intervention.

## 1 Introduction

*Chlamydia pneumoniae* is an obligate intracellular pathogen (1) that is closely linked to both upper and lower respiratory tract infections. It is a major causative agent of community-acquired pneumonia (CAP) (2), with an incidence second only to that of *Streptococcus pneumoniae* and *Mycoplasma pneumoniae*. The general population is broadly susceptible to *Chlamydia pneumoniae* infection. In communal settings such as university dormitories, correctional facilities, hospitals, long-term care institutions, military training camps, and schools, the risk of exposure to this pathogen is markedly elevated. Although surveillance data from multiple centers during the COVID-19 pandemic indicated a decline in the detection rate of *Chlamydia pneumoniae* infection, since the latter half of 2023, a statistically significant resurgence has been documented across many regions worldwide (2–4).

*Chlamydia pneumoniae* pneumonia is also classified as a common type of atypical pneumonia. Compared with typical bacterial pneumonias, such as those caused by *Streptococcus pneumoniae*, atypical pneumonia requires distinctly different therapeutic approaches. Moreover, several studies have suggested that *Chlamydia pneumoniae* infection may be linked to various chronic conditions, particularly atherosclerosis and Alzheimer’s disease, with persistent infection potentially exacerbating these disorders (5, 6). In addition, patients with *Chlamydia pneumoniae* pneumonia may experience reinfection or chronic infection, and prolonged courses of antimicrobial therapy are sometimes necessary for complete eradication (7). Therefore, early and accurate diagnosis of *Chlamydia pneumoniae* pneumonia is of considerable clinical importance.

The diagnosis of *Chlamydia pneumoniae* pneumonia requires multiple complementary approaches, with serological testing remaining an important auxiliary diagnostic method in clinical practice. Although traditional serological testing has a relatively long detection cycle and limited sensitivity and specificity of single indicators, dynamic monitoring of antibody titer changes (e.g., IgM antibody seroconversion or a 4-fold or greater increase in IgG antibody titer) can effectively identify recent infection or acute exacerbation. It is particularly suitable for primary medical institutions lacking invasive sampling conditions or as a supplementary diagnostic scheme in scenarios where metagenomic next-generation sequencing (mNGS) cannot be performed. Notably, Guarino et al. (8) found that patients coinfected with *Chlamydia pneumoniae* and SARS-CoV-2 had lower partial pressure of oxygen and a higher rate of ICU admission, highlighting the importance of early accurate identification. This study confirmed that serological testing is a key method for screening coinfections, further verifying its important role in epidemiological investigations and retrospective diagnoses.

mNGS, based on high-throughput sequencing technology, has been increasingly used in the detection of *Chlamydia pneumoniae* pneumonia due to its high sensitivity and specificity. However, it has strict requirements for respiratory specimens: the detection rate from nasopharyngeal swabs or sputum is not high (7), and invasive respiratory specimens such as bronchoalveolar lavage fluid are usually required, resulting in low patient acceptance.

Clinically, there is a high expectation that imaging examinations, especially CT scans, can provide diagnostic evidence for *Chlamydia pneumoniae* pneumonia. However, few studies have focused on the CT imaging characteristics of *Chlamydia pneumoniae* pneumonia, with most focusing on analyzing its clinical and laboratory features (9, 10). This study adopted a retrospective analysis method, collecting clinical data and chest CT imaging data of 42 patients with *Chlamydia pneumoniae* pneumonia diagnosed in our hospital. For the first time, CT images were grouped and compared according to the time of disease onset, aiming to analyze the clinical and imaging features of the disease, improve the diagnostic level of the disease, especially the exploration of early imaging features, and provide more targeted guidance for clinical diagnosis and treatment.

## 2 Materials and methods

### 2.1 Clinical data

Clinical data of patients diagnosed with *Chlamydia pneumoniae* pneumonia via metagenomic next-generation sequencing (mNGS) were retrospectively obtained from the Sichuan Provincial Corps Hospital of the Chinese People’s Armed Police Force between August 2022 and August 2025.

The inclusion criteria were as follows: (1) meeting the diagnostic criteria for community-acquired pneumonia (CAP) outlined in the 2016 Chinese Guidelines for the Diagnosis and Treatment of Adult CAP (11); (2) submission of bronchoalveolar lavage fluid (BALF) samples in which *Chlamydia pneumoniae* sequences were detected by mNGS; (3) availability of pre-treatment plain chest CT scan data.

The exclusion criteria were: (1) Detection of other pathogens besides *Chlamydia pneumoniae* by mNGS, making it impossible to rule out pulmonary infections caused by other pathogens; (2) Immunocompromised patients; (3) Poor-quality chest CT images that precluded accurate assessment of imaging findings.

Data on patients’ epidemiological history, comorbidities, demographics, clinical manifestations, laboratory results at admission, chest CT findings at admission, and follow-up chest CT findings after treatment were extracted from the hospital’s electronic medical record system. The laboratory parameters primarily included white blood cell count, neutrophil percentage, and C-reactive protein (CRP) levels, and normalized sequence count of *Chlamydia pneumoniae* by mNGS.

### 2.2 Examination methods

All 42 patients underwent non-contrast chest CT examinations using a GE Discovery CT750HD scanner. During the examination, patients were placed in the supine position, and scanning was performed at full inspiration during breath-hold. The scanning range extended from the lung apices to the diaphragmatic bases.

The scanning parameters were as follows: spiral scanning mode; tube voltage, 120 kV; tube current, 100–200 mAs; field of view (FOV), 35 × 35 cm; matrix, 512 × 512; pitch, 0.984–1.2. After acquisition of raw CT data, images were reconstructed using a standard algorithm with a slice thickness of 5 mm, and additional thin-section reconstructions (1 mm thickness, no interval) were generated using a high-resolution algorithm.

### 2.3 Imaging analysis

All chest CT images were independently reviewed by two radiologists, each with over 10 years of experience in thoracic imaging. In cases of disagreement, a third radiologist with over 20 years of experience in thoracic imaging participated in the review, and a consensus was reached through discussion.

The high-resolution CT (HRCT) features were assessed, including:

1. Main intrapulmonary lesion patterns:
  - Lobular pneumonia pattern (bronchial wall thickening with or without surrounding patchy consolidation or ground-glass opacity [GGO]).
  - Lobar pneumonia pattern (lobar or segmental patchy consolidation or GGO with air bronchogram).
  - Nodular pattern (single or multiple round-like patchy or mass-like consolidation, with or without GGO).
2. Associated signs: cavitation or necrosis, interlobular and intralobular septal thickening, crazy-paving pattern, halo sign, reversed halo sign, tree-in-bud and reversed tree-in-bud nodules, and lobular sparing.
3. Extent of lobe involvement: single-lobe versus multi-lobe distribution.
4. Lesion distribution: predominantly upper lung (lesions mainly above the tracheal carina), predominantly lower lung (lesions mainly below the tracheal carina), or diffuse distribution.
5. Extrapulmonary findings: mediastinal or hilar lymphadenopathy and pleural effusion.

### 2.4 Statistical analysis

Statistical analyses were performed using SPSS version 26.0. Categorical variables were summarized as counts and percentages; continuous variables with a normal distribution were presented as mean ± standard deviation, while those with a skewed distribution were presented as median and interquartile range. Fisher’s exact test was used to compare imaging features of intrapulmonary lesions across different stages of disease onset.

The retrospective analysis was approved by the ethics committee of Sichuan Provincial Corps Hospital (Approval No.: 2025–04). The requirement for informed consent was waived.

## 3 Results

### 3.1 Main clinical manifestations

A total of 42 patients with *Chlamydia pneumoniae* pneumonia were included, comprising 39 males (92.9%) and 3 females (7.1%). The patients’ ages ranged from 16 to 61 years, with a mean of 25.8 ± 8.6 years. Thirty patients (71.4%) had a history of residence in closed communities.

The clinical manifestations were: fever in 25 patients (59.5%) with peak temperatures ranging from 37.6 °C to 39.4 °C; sore throat in 26 (61.9%); cough in 30 (71.4%); expectoration in 27 (64.3%); myalgia in 11 (26.2%); fatigue in 10 (23.8%); and neurological symptoms—specifically headache and dizziness—in 10 (23.8%).

Laboratory findings: The median white blood cell count was 9.46 × 10^9^/L, with 30 patients (71.4%) within the normal range and 12 (28.6%) showing mild elevation; the median neutrophil percentage was 72.6%, with 10 patients (23.8%) elevated; the median CRP level was 49.9 mg/L, with 21 patients (50.0%) elevated and 6 (14.3%) exceeding 100 mg/L; the median normalized sequence count of *Chlamydia pneumoniae* by mNGS was 21,675. Clinical Data of Patients with *Chlamydia Pneumoniae* Pneumonia are summarized in Table 1.

**Table 1:** Clinical data of patients with *Chlamydia Pneumoniae* pneumonia / *n* (%).

Items	Results
Age [*M* (*P_25_ ~ P_75_*)] / years	23 (20 ~ 54)
Male [*n* (%)]	39 (92.9%)
Symptoms [*n* (%)]	
Fever	25 (59.5%)
Sore throat discomfort	26 (61.9%)
Cough	30 (71.4%)
Sputum production	27 (64.3%)
Myalgia	11 (26.2%)
General fatigue	10 (23.8%)
Headache, dizziness	10 (23.8%)
White blood cell count [*M* (*P_25_ ~ P_75_*)] / ×10^9^ /L	9.46 (8.62 ~ 11.32)
Neutrophil percentage [*M* (*P_25_ ~ P_75_*)] / %	72.6 (64.3 ~ 89.9)
C - reactive protein [*M* (*P_25_ ~ P_75_*)] / mg·L^−1^	49.9 (0.50 ~ 90.20)
normalized sequence count of *Chlamydia pneumoniae* by mNGS[*M* (*P_25_ ~ P_75_*)]	21,675 (7,865 ~ 84,651)

### 3.2 Imaging findings

A total of 59 chest CT examinations were performed, comprising 42 baseline scans and 17 follow-up scans. All scans were categorized into two groups according to disease duration: scans acquired within 7 days after symptom onset were assigned to the early-stage group (*n* = 29), whereas those obtained between >7 and <30 days were assigned to the middle–late-stage group (*n* = 30). The principal imaging characteristics are summarized in Table 2.

**Table 2:** CT imaging findings of *Chlamydia pneumoniae* pneumonia / *n* (%).

CT Signs	Early-stage group (*n* = 29)	Middle-late stage group (*n* = 30)	*p*
Lesion pattern			
Lobar pattern	5 (17.2%)	7 (23.3%)	0.008
Lobular pattern	16 (55.2%)	23 (76.7%)	
Nodular-patchy pattern	8 (27.6%)	0 (0)	
Lesion extent			
Single lobe	20 (69.0%)	23 (76.7%)	0.506
Multiple lobes	9 (31.0%)	7 (23.3%)	
Lesion distribution by level			
Predominantly upper lung	10 (34.5%)	10 (33.3%)	0.926
Predominantly lower lung	19 (65.5%)	20 (66.7%)	
Diffuse distribution	0 (0)	0 (0)	
Accompanying signs			
Interlobular septal thickening	3 (10.3%)	6 (20.0%)	0.302
Intralobular septal thickening	1 (3.4%)	6 (20.0%)	0.049
Crazy-paving pattern	1 (3.4%)	2 (3.7%)	0.595
Halo sign	25 (86.2%)	21 (70.0%)	0.411
Reversed halo sign	1 (3.4%)	6 (20.0%)	0.087
Centrilobular nodules	23 (79.3%)	20 (66.7%)	0.275
Tree-in-bud sign	14 (48.3%)	16 (53.3%)	0.698
Reversed tree-in-bud sign	17 (58.6%)	10 (33.3%)	0.051
Lobular sparing	19 (65.5%)	7 (23.3%)	0.001
Pleural effusion	2 (6.9%)	8 (26.7%)	0.043
Lymphadenopathy	2 (6.9%)	4 (13.3%)	0.413
Lesion necrosis/cavitation	0 (0)	0 (0)	
Bronchial wall thickening	20 (69.0%)	24 (80.0%)	0.33

#### 3.2.1 Main imaging features of intrapulmonary lesions

##### 3.2.1.1 Early-stage group

**Figure 1:**
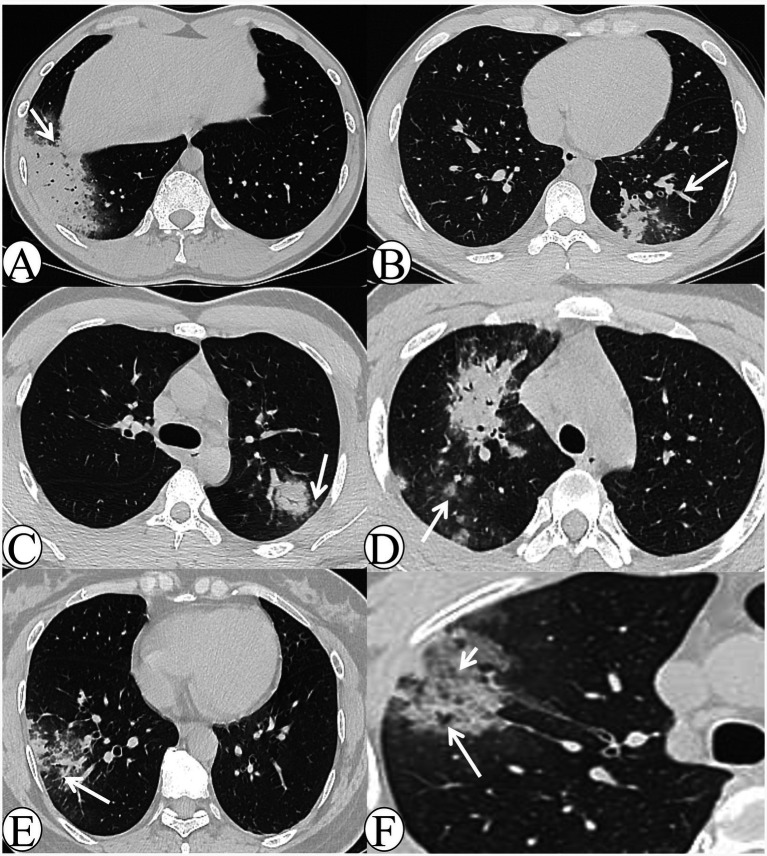
The CT images of early-stage *Chlamydia pneumoniae* pneumonia. **(A)** A 24-year-old male patient presented with fever, productive cough, and headache persisting for 5 days. Axial lung window CT demonstrates extensive consolidation in the right lower lobe with an air bronchogram (white arrow) and a visible peripheral halo sign. **(B)** A 21-year-old male patient presented with fever, pharyngeal hyperemia, and myalgia persisting for 6 days. Axial lung window CT demonstrates bronchial wall thickening in the left lower lobe, accompanied by patchy consolidation and ground-glass opacity (GGO), together with distal tree-in-bud and centrilobular nodules (white arrow). **(C)** A 21-year-old male patient presented with chills and fever for 1 day. Axial lung window CT demonstrates a round-like patch in the left upper lobe, accompanied by an air bronchogram and surrounding ill-defined centrilobular nodules (white arrow). **(D)** A 16-year-old male patient presented with cough and expectoration for 5 days. Axial lung window CT demonstrates multiple mass-like and nodular-patchy consolidations with a halo sign in the right upper lobe (white arrow). **(E)** A 45-year-old female patient presented with chills and fever for 5 days. Axial lung window CT demonstrates peribronchial patchy consolidation and ground-glass opacity (GGO) in the right lower lobe, with visible interlobular septal thickening and lobular sparing (white arrows). **(F)** A 29-year-old male patient presented with fever and cough for 4 days. Axial lung window CT demonstrates patchy consolidation and ground-glass opacity (GGO) in the right upper lobe, with visible intralobular septal thickening (white arrowhead) and a reversed tree-in-bud sign (white arrow).

Chest CT manifestations in the early-stage group included: 5 cases (17.2%) with a lobar pneumonia pattern (Figure 1A), 16 cases (55.2%) with a lobular pneumonia pattern (Figure 1B), and 8 cases (27.6%) with a nodular–patchy pattern (Figures 1C,D). With respect to lobe involvement, 20 cases (69.0%) exhibited single-lobe involvement, whereas 9 cases (31.0%) demonstrated multi-lobe involvement. In terms of lesion distribution, 10 cases (34.5%) were predominantly located in the upper lobes, whereas 19 cases (65.5%) were primarily in the lower lobes; no cases demonstrated diffuse involvement. Regarding ancillary signs, 25 cases (86.2%) exhibited the halo sign, whereas only 1 case (3.4%) exhibited the reversed halo sign. Centrilobular nodules were observed in 23 cases (79.3%), and bronchial wall thickening in 20 cases (69.0%). Additionally, lobular sparing (Figure 1E) was present in 19 cases (65.5%), the reversed tree-in-bud sign (Figure 1F) in 17 cases (58.6%), and the tree-in-bud sign in 14 cases (48.3%). Interlobular septal thickening (Figure 1E) was noted in 3 cases (10.3%), and intralobular septal thickening (Figure 1F) in 1 case (3.4%). No necrosis or cavitation was detected in any patient.

##### 3.2.1.2 Middle–late-stage group

**Figure 2:**
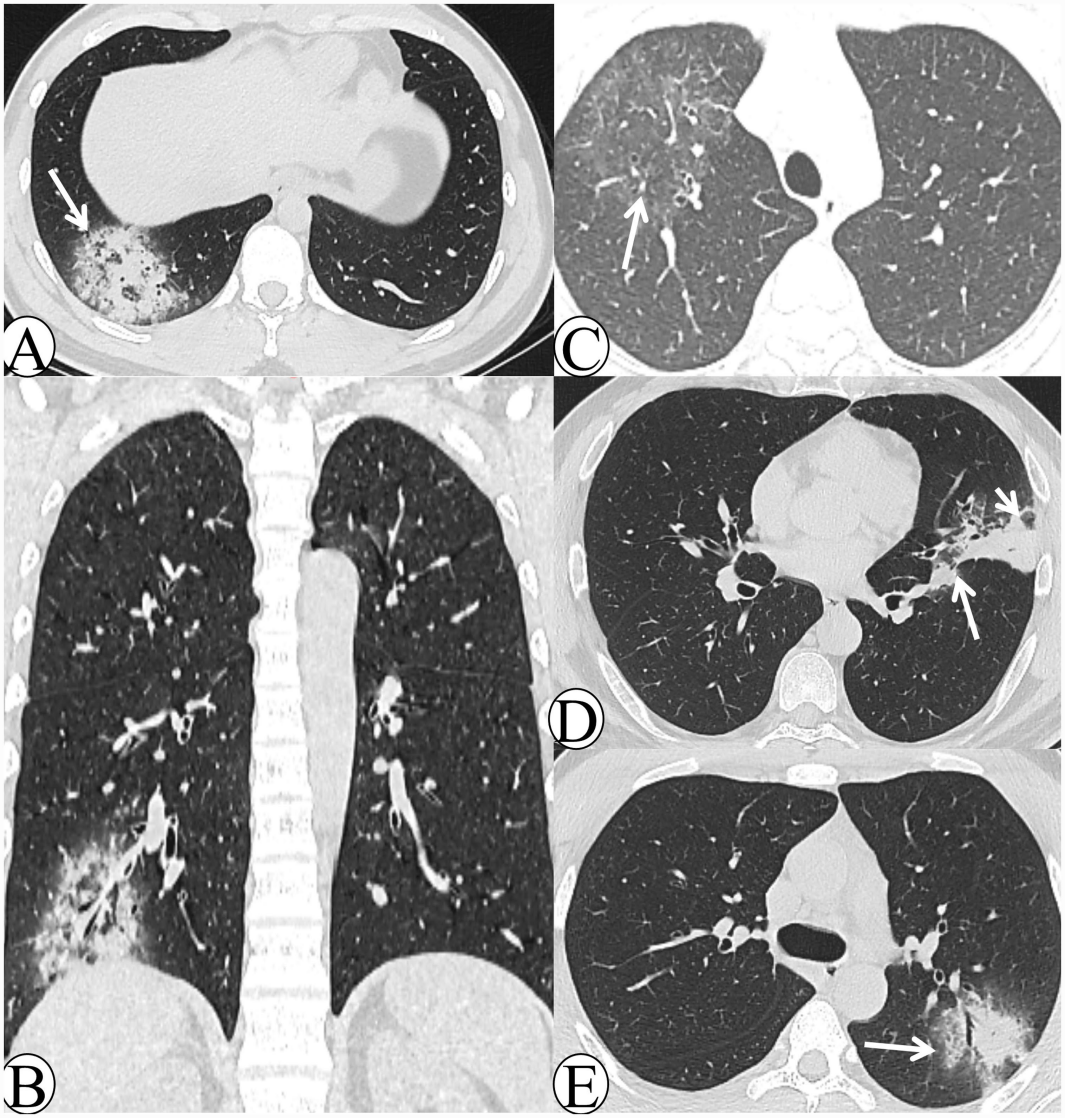
The CT images of the middle–late-stage *Chlamydia pneumoniae* pneumonia. **(A)** A 29-year-old male patient presented with cough and expectoration for 14 days. Axial lung window CT demonstrates mass-like consolidation in the right lower lobe with peripheral halo sign changes, and multiple reversed tree-in-bud signs are visible within the lesion (white arrow). **(B)** The same patient in image A. Coronal lung window CT demonstrates that the lesion exhibits a reversed halo sign appearance. **(C)** A 16-year-old male patient presented with cough and expectoration for 5 days. Follow-up axial lung window CT after 14 days of treatment demonstrates significant resolution of the lesion, which exhibits a faint reversed halo sign (white arrow). **(D)** A 47-year-old male patient presented with cough and expectoration for 20 days. Axial lung window CT demonstrates patchy consolidation in the left upper lobe, with lobular sparing beneath the pleura (white arrowhead) and along the interlobar fissure (white arrow). **(E)** A 21-year-old male patient presented with chills and fever for 1 day. Follow-up axial lung window CT obtained after 8 days of symptomatic treatment demonstrates peribronchial patchy consolidation and ground-glass opacity (GGO) in the left upper lobe, with visible intralobular septal thickening (white arrow).

In the middle–late-stage group, chest CT revealed 7 cases (23.3%) with a lobar pneumonia pattern and 23 cases (76.7%) with a lobular pneumonia pattern; no cases demonstrated a nodular–patchy pattern. Regarding lobe involvement, 23 cases (76.7%) exhibited single-lobe involvement, while 7 cases (23.3%) demonstrated multi-lobe involvement. In terms of lesion distribution, 10 cases (33.3%) were predominantly located in the upper lobes and 20 cases (66.7%) in the lower lobes; none showed diffuse involvement. Ancillary signs included the halo sign in 21 cases (70.0%) (Figure 2A) and the reversed halo sign in 6 cases (20.0%) (Figures 2B,C). Centrilobular nodules were observed in 20 cases (66.7%), and bronchial wall thickening in 24 cases (80.0%). Lobular sparing (Figure 2D) was noted in 7 cases (23.3%), the reversed tree-in-bud sign (Figure 2A) in 10 cases (33.3%), and the tree-in-bud sign in 16 cases (53.3%). Interlobular septal thickening occurred in 6 cases (20.0%) and intralobular septal thickening (Figure 2E) in 6 cases (20.0%). Necrosis or cavitation was absent in all cases.

#### 3.2.2 Extrapulmonary imaging findings

In the early-stage group, chest CT demonstrated hilar or mediastinal lymphadenopathy in 2 cases (6.9%) and small pleural effusions in 2 cases (6.9%). In the middle–late-stage group, chest CT demonstrated hilar or mediastinal lymphadenopathy in 4 cases (13.3%) and small pleural effusions in 8 cases (26.7%).

## 4 Discussion

### 4.1 Clinical characteristics

*Chlamydia pneumoniae* was first isolated in 1965 from conjunctival specimens of a child in Taiwan (12). It exhibits a biphasic life cycle with two morphological forms: the infectious elementary body (EB), which differentiates into the metabolically active, replicative reticulate body (RB) upon host cell invasion. The RB proliferates via binary fission within host cell inclusions and reverts to EBs, which are released to infect new cells—enabling immune evasion and persistent infection (12).

*Chlamydia pneumoniae* pneumonia is a well-recognized subtype of community-acquired pneumonia (CAP), typically presenting with mild clinical manifestations and rare severe cases (10). It infects all age groups but predominantly affects school-aged children, adolescents, and young adults, with occasional outbreaks in confined settings (13, 14). Consistent with previous epidemiological reports, the 42 enrolled patients had a mean age of 25.83 ± 8.59 years, were predominantly young to middle-aged males, and mostly resided in closed communities. The prognosis of *Chlamydia pneumoniae* infection is favorable, with a reported mortality rate below 2% (13).

*Chlamydia pneumoniae* infects the respiratory tract by adhering to and invading epithelial cells; its intracellular proliferation induces cell death and inflammatory responses, leading to pneumonia. The predominant clinical manifestations in this cohort were cough (71.4%), expectoration (64.3%), and sore throat (56.5%)—findings consistent with prior studies (12). Additional symptoms included fever (59.5%), myalgia (26.2%), general fatigue (23.8%), and neurological symptoms (headache, dizziness; 23.8%). Laboratory findings were characterized by normal or mildly elevated white blood cell counts, increased neutrophil percentages, and normal or mildly elevated C-reactive protein (CRP) levels, which align with previous reports (14, 15).

### 4.2 Imaging characteristics

In this study, chest CT scans were, for the first time, categorized into two groups (early stage and middle-to-late stage) based on the time of symptom onset, to systematically analyze and statistically assess imaging features. The imaging findings are summarized as follows:

#### 4.2.1 Imaging patterns

The lobular pneumonia pattern represented the predominant finding in both the early-stage and middle-to-late-stage groups (55.2 and 76.7%, respectively). Furthermore, the nodular–patchy pattern was exclusively detected in the early-stage group (27.6%) and was absent in the middle-to-late-stage group. The time of symptom onset for cases with this pattern ranged from 1 to 4 days. These findings suggest that in the early stage of disease, the primary sites of involvement are the alveoli and bronchioles, with some lesions exhibiting nodular–patchy changes. As the disease progresses, inflammation spreads to more proximal bronchi and across alveolar structures, ultimately evolving into a bronchopneumonia or other patterns. This distinctive imaging feature has not been previously documented. We propose that the presence of a nodular–patchy pattern indicates the early or ultra-early stage of the disease, and that all cases of *Chlamydia pneumoniae* pneumonia may initially present with this manifestation. However, this pattern is transient and rapidly progresses to other manifestations. This hypothesis warrants further validation in studies with larger sample sizes.

#### 4.2.2 Lesion location

Both the early- and middle-to-late-stage groups were characterized by predominant single-lobe involvement (69.0 and 76.7%, respectively) and lower-lung predilection (65.5 and 66.7%, respectively), findings that are consistent with previous reports (15). No cases exhibited diffuse distribution, likely attributable to the predominance of mild disease and the absence of severe cases in this cohort.

#### 4.2.3 Associated signs

The halo sign represented the predominant radiologic feature in both the early- and middle-to-late-stage groups (86.2 and 70.0%, respectively). The presence of the halo sign reflects inflammatory exudation or hemorrhage, typically corresponding to an acute pathological process (16). Previous studies have reported that *Chlamydia pneumoniae* can transition into a persistent infectious state (e.g., aberrant bodies), evade antimicrobial therapy and host immune responses via morphological and metabolic alterations, and thereby contribute to both acute disease progression and potential chronic sequelae (17). Therefore, the halo sign—indicative of acute exudation—may be observed in both early and middle-to-late stages; however, this interpretation warrants further confirmation in studies with larger sample sizes.

The next most frequently observed features included bronchial wall thickening, centrilobular nodules, lobular sparing, as well as reversed tree-in-bud and tree-in-bud signs. Among these, the incidence of lobular sparing was significantly higher in the early-stage group compared with the middle-to-late-stage group (*p* = 0.001). These findings suggest that during the early stage, the bronchioles and alveoli are primarily involved, whereas in later stages, as lesions merge into lobular or lobar pneumonia, lobular involvement becomes more extensive, resulting in decreased lobular sparing. Additionally, the presence of both reversed tree-in-bud and tree-in-bud signs further supports the notion that the disease predominantly affects the bronchioles and alveoli (18).

Regarding interlobular (10.3% vs. 20.0%) and intralobular septal thickening (3.4% vs. 20.0%), the incidence of intralobular septal thickening was significantly higher in the middle-to-late-stage group compared with the early-stage group (*p* = 0.049). This finding suggests that the disease can involve the pulmonary interstitium, producing imaging manifestations that resemble those of viral pneumonias (e.g., influenza, COVID-19, adenovirus) and intracellular bacterial infections (e.g., *Legionella*, *Chlamydia psittaci*) (18).

The reversed halo sign was observed in one case in the early-stage group and six cases in the middle-to-late-stage group, with no statistically significant difference between groups. The reversed halo sign occurs in a variety of conditions, including infectious and non-infectious inflammatory diseases as well as neoplasms (19), and its underlying mechanisms are heterogeneous. It has also been frequently reported in atypical pneumonias. In the context of *Chlamydia pneumoniae* pneumonia, we hypothesize that the reversed halo sign may result from lesion spread through Kohn pores, accompanied by earlier absorption of the central portion compared with the periphery. In the middle-to-late stage, lesions with a reversed halo sign often demonstrate relatively well-defined margins and partial fibrotic changes, findings that presumably indicate immune-mediated repair processes. Whether this sign can serve as a prognostic indicator requires confirmation in studies with larger sample sizes.

Necrosis and cavitation are rare manifestations of *Chlamydia pneumoniae* pneumonia. No cases of necrosis or cavitation were identified in this study, consistent with previous reports, likely reflecting the virulence characteristics of *Chlamydia pneumoniae*.

Previous studies have consistently shown that *Chlamydia pneumoniae* pneumonia is rarely complicated by pleural effusion or lymphadenopathy (14, 15). In the present cohort, pleural effusion was observed in 6.9% of early-stage cases and 26.7% of middle-to-late-stage cases, rates comparable to those reported by Cao Xiaobei et al. (20) (13.7%) and consistent with previous observations. Extrapulmonary manifestations, including hilar and mediastinal lymphadenopathy (6.9 and 13.3%), were also consistent with prior studies.

#### 4.2.4 Differential diagnosis

The clinical diagnosis of *Chlamydia pneumoniae* pneumonia requires strict differentiation from pneumonias caused by other pathogens, with key discriminative features summarized as follows:

1. *Mycoplasma pneumoniae* pneumonia: Predominantly affects children and young to middle-aged adults, presenting with fever, dry cough, fatigue, and the hallmark of “more severe imaging than clinical manifestations” (21). Imaging mainly shows a lobular pneumonia pattern with bronchial wall thickening, patchy ground-glass opacity (GGO), and a typical “tree-mist” appearance. The rarity of tree-in-bud sign and pulmonary interstitial involvement distinguishes it from *Chlamydia pneumoniae* pneumonia.
2. *Chlamydia psittaci* pneumonia: Prevalent in middle-aged and elderly individuals, mostly with avian/poultry exposure history. High-risk factors include male gender, smoking, alcoholism, immunosuppression, and chronic cardiopulmonary diseases (22). It often involves multiple systems (e.g., headache, myalgia, delirium) and is prone to complications like electrolyte disturbances. Imaging is dominated by mixed consolidation and GGO with blurred boundaries, accompanied by common intralobular septal thickening (fine reticular sign) and reversed halo sign, while necrosis, cavitation, and tree-in-bud sign are rare (23). Differentiation from *Chlamydia pneumoniae* pneumonia relies on the latter’s frequent halo sign/tree-in-bud sign, predominant lobular pattern, younger age at onset, lack of avian/poultry exposure, and rare severe cases.
3. Viral pneumonia: Includes adenovirus, COVID-19, and influenza-related pneumonias. Adenovirus pneumonia is common in infants/adolescents with potential closed-community outbreaks; influenza/COVID-19 exhibit seasonal epidemiology and universal susceptibility, presenting with high fever. Imaging features: Adenovirus pneumonia shows centrilobular nodules with fused consolidation, GGO, and septal thickening (24); COVID-19 is dominated by subpleural patchy GGO/consolidation (25); influenza pneumonia involves bronchovascular bundles with mucus impaction and pleural effusion (26). Early *Chlamydia pneumoniae* pneumonia (nodular-patchy consolidation with halo sign) is radiologically indistinguishable from these viral pneumonias, but its progression to lobular pneumonia with bronchial wall thickening differs. Comprehensive judgment requires epidemiological history (e.g., closed-community exposure) and etiological results (e.g., viral nucleic acid testing, mNGS).
4. *Streptococcus pneumoniae* pneumonia: Common in young to middle-aged adults and the elderly, presenting with high fever, chills, and productive cough. Laboratory tests show marked leukocytosis. Imaging is characterized by large lobar/segmental consolidation with frequent air bronchograms, while tree-in-bud sign, bronchial wall thickening, and halo sign are extremely rare—distinct from *Chlamydia pneumoniae* pneumonia.
5. Pneumocystis jiroveci pneumonia: Restricted to immunocompromised patients, presenting with hypoxemia, wheezing, and dry cough. Laboratory tests reveal elevated fungal G test results. Imaging shows extensive bilateral GGO (with/without small vesicles) and subpleural sparing (27), differing from *Chlamydia pneumoniae* pneumonia clinically and radiologically.
6. Invasive pulmonary aspergillosis: Vascular involvement may mimic *Chlamydia pneumoniae* pneumonia (nodular-patchy consolidation with halo sign). However, most patients have an immunosuppression history, and post-treatment lesions may develop cavitations (e.g., crescent sign) (28), enabling differentiation.
7. Pulmonary cryptococcosis: Caused by *Cryptococcus neoformans* or C. gattii, occasionally with poultry exposure. Clinical/imaging features depend on host immunity: immunocompetent patients are often asymptomatic with incidentally detected single/multiple nodules (wide pleural attachment, frequent halo sign). Enhanced scanning may show “ghost face”-like necrosis; positive latex agglutination test or cryptococcal antigen detection is diagnostic (29), distinguishing it from *Chlamydia pneumoniae* pneumonia.

This study has certain limitations in its design and implementation, as follows: First, the sample size included in this study is relatively small, with a predominance of male cases, resulting in a gender selection bias. Although subgroup analysis was performed based on the time of symptom onset, the correlations between different imaging patterns, signs, and the disease itself were not further explored, which may have led to the oversight of some potential associated characteristics. Second, this study is a single-center retrospective investigation. Constrained by the study design and regional population characteristics, the generalizability of the research conclusions may be limited. Therefore, reliable clinical features and CT manifestations of *Chlamydia pneumoniae* pneumonia still require further verification and refinement through subsequent large-sample, multi-center prospective studies. Conclusion.

In conclusion, *Chlamydia pneumoniae* pneumonia demonstrates distinctive clinical and radiological features. Clinically, it occurs more often in younger patients, generally presents with mild symptoms, and is prone to outbreaks in closed communities. Laboratory findings usually reveal normal or only slightly elevated white blood cell counts and C-reactive protein levels. Radiologically, the lobular pneumonia pattern predominates, frequently accompanied by halo sign, bronchial wall thickening, centrilobular nodules, tree-in-bud sign, and reversed tree-in-bud sign, whereas necrosis and cavitation are extremely rare. Certain imaging differences exist between early and middle-to-late stages, with the nodular-patchy pattern potentially representing an ultra-early imaging marker. Recognition of this feature may facilitate timely therapeutic interventions.

## Data Availability

The raw data supporting the conclusions of this article will be made available by the authors, without undue reservation.
